# Influence of traumatic brain injury on extracellular tau elimination at the blood–brain barrier

**DOI:** 10.1186/s12987-021-00283-y

**Published:** 2021-10-26

**Authors:** Maxwell Eisenbaum, Andrew Pearson, Arissa Gratkowski, Benoit Mouzon, Michael Mullan, Fiona Crawford, Joseph Ojo, Corbin Bachmeier

**Affiliations:** 1 The Roskamp Institute, 2040 Whitfield Avenue, Sarasota, FL 34243 USA; 2 The Open University, Milton Keynes, UK; 3 James A. Haley Veterans’ Hospital, Tampa, FL USA; 4 Bay Pines VA Healthcare System, Bay Pines, FL USA

**Keywords:** Mural cells, Tau, Pericytes, Endothelial cells, Traumatic brain injury, Blood–brain barrier, Caveolin-1, Angiopoietin-1, Angiopoietin-2, Mfsd2a

## Abstract

Repetitive head trauma has been associated with the accumulation of tau species in the brain. Our prior work showed brain vascular mural cells contribute to tau processing in the brain, and that these cells progressively degenerate following repetitive mild traumatic brain injury (r-mTBI). The current studies investigated the role of the cerebrovasculature in the elimination of extracellular tau from the brain, and the influence of r-mTBI on these processes. Following intracranial injection of biotin-labeled tau, the levels of exogenous labeled tau residing in the brain were elevated in a mouse model of r-mTBI at 12 months post-injury compared to r-sham mice, indicating reduced tau elimination from the brain following head trauma. This may be the result of decreased caveolin-1 mediated tau efflux at the blood–brain barrier (BBB), as the caveolin inhibitor, methyl-β-cyclodextrin, significantly reduced tau uptake in isolated cerebrovessels and significantly decreased the basolateral-to-apical transit of tau across an in vitro model of the BBB. Moreover, we found that the upstream regulator of endothelial caveolin-1, Mfsd2a, was elevated in r-mTBI cerebrovessels compared to r-sham, which coincided with a decreased expression of cerebrovascular caveolin-1 in the chronic phase following r-mTBI (> 3 months post-injury). Lastly, angiopoietin-1, a mural cell-derived protein governing endothelial Mfsd2a expression, was secreted from r-mTBI cerebrovessels to a greater extent than r-sham animals. Altogether, in the chronic phase post-injury, release of angiopoietin-1 from degenerating mural cells downregulates caveolin-1 expression in brain endothelia, resulting in decreased tau elimination across the BBB, which may describe the accumulation of tau species in the brain following head trauma.

## Introduction

Exposure to repetitive head injuries sustained in the military or contact sports has been associated with an increased risk for the development of chronic neurodegenerative diseases, including chronic traumatic encephalopathy (CTE) [34]. Though the underlying mechanism behind the progression of CTE is unclear, the neuropathologic presentation has become apparent through postmortem examination. CTE appears to be a primary tauopathy characterized, in part, by a perivascular accumulation of pathological hyperphosphorylated tau at the depths of cortical sulci [35]. Tau, which primarily supports the functions of microtubules in neurons, has traditionally been viewed as an intracellular protein, but recent research has revealed an important pathological role of extracellular tau in neurodegenerative progression [36]. Furthermore, traumatic brain injury (TBI) results in elevated levels of extracellular tau in the interstitial fluid (ISF) of the central nervous system (CNS) [33], which has been correlated with adverse clinical outcomes [32, 46].

Though extracellular tau has an important role in disease pathogenesis, the mechanisms by which extracellular tau is eliminated from the brain and the influence of TBI on these processes has been largely unexplored. While extracellular solutes can be eliminated from the brain through bulk flow or perivascular pathways, a major route of elimination occurs via transit across the blood–brain barrier (BBB). Microdialysis studies investigating tau movement in brain interstitial fluids have suggested tau may be more dynamically linked to the blood than the cerebrospinal fluid [57, 59], implicating possible interactions with the BBB. Several pathogenic proteins have been shown to cross the BBB [7, 13, 53], including tau [8], but the processes driving BBB tau transit are not fully understood, particularly following trauma to the brain. It has been suggested that degradation and perivascular clearance may be responsible for a greater magnitude of extracellular tau elimination than bulk flow to the CSF [59].

Our recent work found brain vascular mural cells progressively degenerate at chronic time points following repetitive mild traumatic brain injury (r-mTBI) in a mouse model of concussion [45]. Moreover, these effects were associated with reduced cerebrovascular tau uptake in freshly isolated cerebrovessels from r-mTBI animals. Notably, the decrease in tau uptake post-injury coincided with significant reductions in cerebrovascular caveolin-1 levels in the mouse r-mTBI model. Caveolin-1 is an integral membrane component for the formation and function of caveolae-mediated cerebrovascular transcytosis. Additionally, our prior studies observed decreased caveolin-1 expression in cerebrovessels from human TBI brain specimens when compared to non-injured control brains [45]. While few studies have investigated the relationship between tau and cerebrovascular caveolin-1, it has been reported that mice with decreased caveolin-1 expression exhibit elevated levels of total and phosphorylated tau in the brain [10, 25]. Caveolin-1 activity in brain endothelial cells is primarily regulated by the lysolipid transporter Mfsd2a (major facilitator superfamily domain‐containing protein‐2a) [2, 9]. Mfsd2a plays a critical role in maintaining BBB permeability, and it was found that overexpression of Mfsd2a after brain injury can be neuroprotective [63]. While it has been observed that Mfsd2a levels downregulate in the acute phase following brain injury [16, 17], the state of cerebrovascular Mfsd2a at more chronic stages post-injury have not been investigated, not to mention the resulting effect on caveolin-1 levels and tau elimination across the BBB. The goal of this study is to investigate the mechanisms influencing tau elimination at the BBB and determine the chronic effects of repetitive head trauma on these processes.

## Materials and methods

### Materials

Primary human brain vascular pericytes (HBVP) (cat#1200), primary human brain microvascular endothelial cells (HBMEC) (cat#1000), and associated culture reagents were purchased from Sciencell Research Laboratories (Carlsbad, CA, USA). Fibronectin solution (cat#F1141), poly-L-lysine solution (cat#P4707), methyl-β-cyclodextrin (cat#C4555), Thioflavin S (cat#T1892-25G), heparin (cat#H3393-10KU) and Hanks’ balanced salt solution (HBSS) (cat#H8264) were purchased from MilliporeSigma (St. Louis, MO, USA). Lucifer yellow dextran (10 kDa) and the human tau enzyme linked immunosorbent assay (ELISA) (cat#KHB0041) were purchased from Invitrogen Corp. (Carlsbad, CA, USA). Mammalian protein extraction reagent (M-PER) (cat#78505), Halt enzyme inhibitor cocktails (cat#78442), SuperSignal West Fempto Maximum Sensitivity Substrate (cat#PI34095), and the bicinchoninic acid (BCA) protein assay (cat#23225) were purchased from ThermoFisher Scientific (Waltham, MA, USA). The ELISA kits for mouse Major facilitator superfamily domain containing 2 (Mfsd2a) (cat#LS-F17827-1), and human Mfsd2a (cat#LS-F17826-1) were purchased from LifeSpan BioSciences, Inc. (Seattle, WA, USA). The ELISA kit for mouse caveolin-1 (cat#MBS721447) was purchased from MyBioSource, Inc. (San Diego, CA, USA). The ELISA kit for mouse angiopoietin-1 (Ang-1) (cat#NBP2-62857) was purchased from Novus Biologicals (Littleton, CO, USA). The ELISA kit for mouse angiopoietin-2 (Ang-2) (cat#MANG20) was purchased from R&D Systems (Minneapolis, MN, USA). Recombinant human tau-441 (rhtau) (cat#T-1001-2) was purchased from rPeptide (Watkinsville GA, USA). Recombinant biotin-labeled human tau-441 (cat#T08-54BN), and recombinant DYRK1A-phosphorylated and biotin-labeled human tau (cat#T08-50RNB) were purchased from SignalChem (Richmond, BC, Canada).

### Animals

Both male and female mice [Human tau (hTau) (cat# 005491) and wild-type (C57BL/6) (cat# 000664)] were purchased from the Jackson Laboratory (Bar Harbor, ME, USA). All studies used a mix of male and female mice. The hTau mice express six isoforms of human tau on a C57BL/6 background, but do not express murine tau, as previously described [1]. The hTau genotype was confirmed after purchase using PCR from a tail snip via a third party (Transnetyx, Cordova, TN, USA). In our recent work, we investigated several time points following r-mTBI using the hTau animals to evaluate the association between mural cell dysfunction and tau accumulation in the brain [45]. To better align the current studies with this previous work, we used the same hTau tissue samples from the prior studies to evaluate the chronic effect of r-mTBI on angiopoietin, Mfsd2a, and caveolin-1. All studies used mice housed 3 per cage under standard laboratory conditions (23 ± 1 ℃, 50 ± 5% humidity, and a 12-h light/dark cycle) with free access to food and water throughout the study. All experiments using animals were performed under protocols approved by the Institutional Animal Care and Use Committee (IACUC) of the Roskamp Institute.

### Brain injury protocol

Repetitive mild traumatic brain injury (r-mTBI) was administered using a mouse model of closed head injury as previously characterized by our group [39, 40]. Briefly, after being 1.5 L/min of oxygen and 3% isoflurane, mice had their head shaved and were secured in a mouse stereotaxic apparatus (Stoelting) mounted with an electromagnetic controlled impact device (Leica) and a heating pad to maintain their body temperature. Before impact, a 5 mm blunt metal impactor tip was retracted and positioned midway in relation to the sagittal suture. The injury was triggered using the myNeuroLab controller (Leica) at a strike velocity of 5 m/s, strike depth of 1.0 mm, and a dwell time of 200 ms. Randomly assigned three-month-old mice received 2 injuries per week, approximately 72 h apart, for 3 months (r-mTBI). As a control, sham animals did not receive the brain injury, but were exposed to anesthesia for the same length of time as the injured mice and under the same paradigm (2 exposures per week for 3 months). Mice (hTau) were euthanatized at 24 h, 3 months, 6 months after the final brain injury or anesthesia exposure. The sample sizes for the r-sham and r-mTBI groups were the same for each post-injury time point: 24 h (n = 4), 3 months (n = 7), and 6 months (n = 11). For the intracranial tau injection studies, mice (wild-type) were euthanized at 12 months after the final brain injury. The sample sizes for the r-sham and r-mTBI groups in the intracranial tau injection study were n = 4–8 for each tau species.

### Isolation of brain fractions

The cerebrovasculature was isolated from mouse tissue as characterized and described by our group previously [4]. Briefly, fresh mouse cortices were ground in 5 ml of ice-cold HBSS with 6–8 passes of a Teflon pestle in a glass Dounce homogenizer. A 250 μl aliquot of homogenate was collected with lysis buffer (M-PER) supplemented with phenylmethanesulfonyl fluoride (1 mM) and Halt protease and phosphatase inhibitor cocktail. An equal volume of 40% dextran solution was added to the remaining brain homogenate for a final concentration of 20% dextran and immediately centrifuged at 6000 g for 15 min at 4˚C. This procedure results in a pellet at the bottom of the container (cerebrovasculature) and a compact mass at the top of the solution (parenchyma) separated by a clear dextran interface (soluble fraction, i.e., non-cell associated). The freshly isolated vessels were collected and immediately used for the ex vivo studies described below.

### Tau aggregation

Enriched fractions of low molecular weight aggregated tau were generated as previously described [37]. Briefly, biotin-labeled tau (4.35 µM) was incubated with freshly prepared heparin (1 µM) for 6 h at 37 °C. To monitor aggregation activity, monomeric biotin-labeled tau (4.35 µM) was incubated with or without heparin (1 µM) in the presence of the aggregation indicator dye, Thioflavin S (ThS) (20 µM). ThS fluorescence (excitation 450 nm, emission 510), was analyzed at baseline (0 h) and at 6 h for each group using a microplate spectrofluorometer (Biotek Cytation 3), to determine the change in relative fluorescence units. After confirmation of aggregation, the aggregate enriched solution of biotin-labeled tau was passed through a 100 kDa MWCO filter (Corning) and centrifuged at 14,000 × g for 25 min at 4 °C. The concentrated, aggregate enriched fraction (MW > 100 kDa) was collected and stored at − 80 °C. The filtered fraction of seed competent monomers and dimers (MW < 100 kDa) were subsequently concentrated using a 30 kDa MWCO filter (Corning) and centrifuged at 14,000 × g for 25 min at 4 °C. The final protein concentrations were determined using a biotin-labeled tau ELISA. Misfolding of the seed competent monomeric fraction was confirmed using dot blot. Concentration (50 ng/ml) matched aliquots (1 μl) of monomeric biotin-labeled tau, seed competent biotin-labeled tau, and aggregate enriched biotin-labeled tau, were placed on a nitrocellulose membrane for 30 min at room temperature before blocking in 10% BSA in tris buffered saline with 0.1% Tween-20 (TBS-T) overnight at 4 °C. The membrane was incubated for 1 h in MC1 (MC1, 1:1000 diluted in TBS-T with 5% BSA), vigorously washed with TBS-T, then incubated in horseradish peroxidase-conjugated anti-mouse secondary antibody (goat anti-mouse IgG 1:1000 in TBS-T with 5% BSA) for 1 h at room temperature. After an additional wash in TBS-T, SuperSignal West Femto Maximum Sensitive Substate (ThermoFisher) was used for chemiluminescence detection and signal intensity ratios were quantified with the ChemiDoc TM XRS (Bio-Rad). MC1 is a conformation-dependent tau antibody raised against paired helical filament tau from Alzheimer’s disease brain homogenate, whose reactivity depends on the N terminus (amino acids 7–9) and the C-terminus (amino acids 313–322) [27], and was generously provided by Dr. Peter Davies, The Feinstein Institute for Medical Research, Bronx, NY, USA.

### Tau elimination

For the temporal tau elimination studies, 6 wild-type mice (9 months of age) were anesthetized via inhalation using a 3% isoflurane/oxygen mix and maintained at 37 °C using a homeothermic blanket. mice were stereotaxically injected into the brain with (50 μg/ml) human biotin-labeled tau in 3 μl of PBS (0.5 mm anterior to the bregma, 2 mm lateral to the midline, and 3 mm below the surface of the skull) as per our prior methods [47]. In a separate cohort of mice, 10 kDa lucifer yellow dextran (LyD) (100 mg/ml) was stereotaxically injected into the brain in the same manner as above, to provide context for the tau studies, as LyD does not readily cross the BBB [42]. Mice were euthanized at 10 min, 30 min, 1, 2, 4, 8, and 24 h after the intracranial injection. The brain was harvested, and each hemisphere was homogenized with probe sonication in 500 μl of lysis buffer. The half-life for both biotin-labeled tau and LyD was determined using nonlinear regression and a one phase decay fit (GraphPad Prism 8.0, GraphPad Software, Inc) using the following equation: Y = (Y0 − Plateau) * exp (-K * X) + Plateau. Y0 is the Y value when X (time) is zero and the Plateau is the Y value at infinite times, both of which are expressed in the same units as Y. K is the rate constant, expressed in reciprocal of the X axis time units. If X is in minutes, then K is expressed in inverse minutes. Tau is the time constant, expressed in the same units as the X axis. It is computed as the reciprocal of K. The half-life is in the time units of the X axis. It is computed as ln(2)/K. Lastly, the span is the difference between Y0 and Plateau, expressed in the same units as the Y values [38]. The value at time = 0 (y-intercept) was used as the theoretical initial concentration in the brain and the values at each time point were calculated as a percentage of this initial concentration.

### Tau residence in the brain

Biotin-labeled tau species and 10 kDa lucifer yellow dextran (LyD) were stereotaxically injected into r-sham and r-mTBI wild-type mice (12 months post-injury), in the same manner as the temporal studies above, and euthanatized 2 h after the intracranial injection. The brain homogenate was evaluated for biotin-labeled tau using a modified hTau ELISA (Invitrogen). The ELISA was performed according to the manufacturer’s protocol, using stock biotin-labeled tau as the standard and excluding the 1-h incubation step with a biotin-conjugated primary antibody. Furthermore, each stereotaxic injection included LyD (80 mg/ml) to account for any nonspecific leakage out of the brain, as LyD typically demonstrates low BBB permeability [42]. LyD fluorescence was analyzed using a microplate spectrofluorometer (Biotek Cytation 3). With respect to tau, all samples were evaluated for exogenous biotin-labeled tau remaining in the brain and normalized to LyD for each time point. To assess LyD alone, the amount of LyD at each time point was normalized to total protein content as determined using the BCA assay.

### Caveolin-1 and Mfsd2a expression in r-mTBI mice

Cerebrovessels from hTau r-sham and r-mTBI mice at 24 h, 3 and 6 months post-injury were collected using lysis buffer (M-PER) supplemented with phenylmethanesulfonyl fluoride (1 mM) and Halt protease and phosphatase inhibitor cocktail. The cell lysates were analyzed for caveolin-1 and Mfsd2a by ELISA and normalized to total protein content using the BCA protein assay.

### Tau uptake and caveolin inhibition ex vivo

Freshly isolated cerebrovessels from naïve wild-type mice (9 months of age) were pre-treated with methyl-β-cyclodextrin (0, 1, and 10 mM) for 30 min at 37˚C followed by treatment with 5 ng/ml rhtau for 1 h at 37 ℃. Following the treatment period, the extracellular media was removed, and the cerebrovessels were washed with ice-cold HBSS. Cell lysates were collected using lysis buffer (M-PER) supplemented with phenylmethanesulfonyl fluoride (1 mM) and Halt protease and phosphatase inhibitor cocktail. The cell lysates were analyzed for total tau by ELISA and normalized to total protein content using the BCA protein assay.

### Angiopoietin-1 modulates HBMEC expression of Mfsd2a in vitro

Fully confluent HBMECs were treated with 2.5 ng/ml of Ang-1 or a vehicle in ECM for 24 h. Cells were washed with HBSS and cell lysates were collected using lysis buffer as described previously. The cell lysates were analyzed for Mfsd2a by ELISA and normalized to total protein content using the BCA protein assay.

### Cerebrovascular angiopoietin 1/2 secretion ex vivo

Freshly isolated cerebrovessels from r-sham and r-mTBI mice 6 months post-injury were incubated in ECM for 72 h at 37˚C. Following the incubation, the extracellular media was collected, the cerebrovessels were washed in ice-cold HBSS, then collected in lysis buffer as described previously. The extracellular media was evaluated for secretion of Ang-1 and Ang-2 by ELISA and normalized to the corresponding cell lysate total protein content using the BCA protein assay.

### In vitro BBB assay

Using HBVP and HBMEC cells, a contact coculture version of our previously characterized in vitro BBB model (Bachmeier 2010) was used to evaluate tau transcytosis. Briefly, HBVP were seeded at 25,000 cells/cm^2^ onto the exterior portion of poly-L-lysine coated 24-well 0.4 μm-pore membrane inserts. One hour after HBVP seeding, HBMEC were seeded at 50,000 cells / cm^2^ onto the interior membrane of the fibronectin-coated insert to establish a polarized monolayer. The layer of cells separates this system into an apical (“blood” side) and basolateral (“brain” side) compartment. The basolateral compartment was exposed to monomeric or aggregated biotin-labeled tau (200 ng/ml) in the presence or absence of MβCD (10 mM), while fresh media was placed in the apical compartment. The inserts containing media were exposed to the wells containing biotin-labeled tau and incubated at 37 °C. The basolateral compartment was sampled at time 0 to establish the initial concentration of biotin-labeled tau. Samples were collected from the apical compartment at 0, 30, and 60 min to assess the rate of biotin-labeled tau transcytosis across the cell monolayer (basolateral-to-apical) and analyzed for biotin-labeled tau using a modified hTau ELISA. Furthermore, each basolateral compartment was exposed to a known paracellular marker, 10 kDa lucifer yellow dextran (LyD 10 µM), to monitor cellular integrity and/or nonspecific permeability, as we previously described [5]. The apparent permeability (Papp) was determined using the equation Papp = 1/AC_0_ * (dQ / dt), where A represents the surface area of the membrane, C0 is the initial concentration of biotin-labeled tau in the basolateral compartment, and dQ/dt is the amount of biotin-labeled tau appearing in the apical compartment in the given time period [3].

### Statistical analysis

Randomization and blinding procedures were employed. Quantitative data were plotted as mean ± standard deviation. Statistical analysis was performed using GraphPad Prism 8.0 (GraphPad Software, Inc). The Shapiro–Wilk test was completed to assess normality. Tau uptake was evaluated for significance by ANOVA and the Bonferroni post hoc test with multiple comparisons. Tau elimination, as well as caveolin-1 and Mfsd2a expression were evaluated for significance as determined by two-way ANOVA Bonferroni post hoc test with multiple comparisons. For comparisons between two groups including tau transcytosis, angiopoietin secretion and stimulation studies, statistical significance was analyzed using a two-tailed unpaired Student’s t-test or a Mann–Whitney U test. For all analyses, a p value of ≤ 0.05 was considered statistically significant.

## Results

### Tau residence time in the brain

**Fig. 1:**
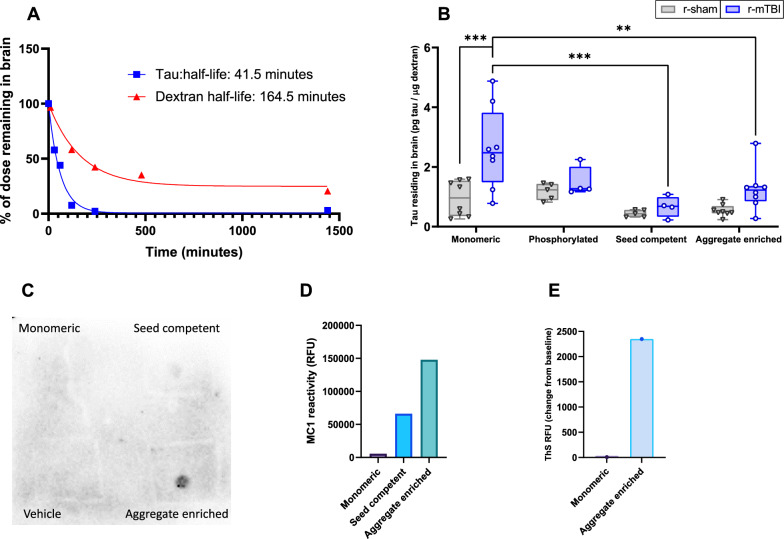
Effect of r-mTBI on the elimination of exogenous tau species from the brain. **A** The time course of tau elimination from the brain was established by examining biotin-labeled tau (n = 6) and 10 kDa LyD (n = 5) levels in the brain at various time points following intracranial injection into naïve wild-type mice (9 months of age). Biotin-labeled tau content was analyzed using an ELISA and LyD was analyzed via fluorescence. The half-life for both biotin-labeled tau and LyD were determined using nonlinear regression and a one phase decay fit. **B** Following intracortical injection in r-sham (n = 5–8) and r-mTBI mice (n = 4–8) (12 months post-injury), the amount of exogenous biotin-labeled tau species residing in the brain was determined at 2 h post-injection. For each injection, biotin-labeled tau was co-injected with LyD. Biotin-labeled tau content was analyzed using an ELISA while LyD was analyzed via fluorescence. Values represent mean ± SD (n = 4–8) and are expressed as pg of tau per μg of LyD. **P < 0.01, ***P < 0.001 as compared to r-sham as determined by a two-way ANOVA and Bonferroni post-hoc test. **C**–**E** In characterizing each tau species, biotin-labeled tau (4.35 µM) was incubated with heparin (1 µM) for 6 h at 37 °C to induce aggregation. The aggregates were separated using a 100 kDa molecular weight cutoff filter into aggregate enriched (tau > 100 kDa) or seed competent (tau < 100 kDa) fractions. **C** The conformational status of the seed competent (50 ng/ml) and aggregate enriched (50 ng/ml) biotin-labeled tau fractions were compared to monomeric (50 ng/ml) biotin-labeled tau via dot blot using an MC1 antibody. **D** Quantitative analysis of MC1 immunoreactivity in the dot blot. **E** Heparin-induced in vitro aggregation of monomeric biotin-labeled tau (4.35 µM) was assessed using the indicator dye ThS. ThS fluorescence was measured in the presence of monomeric biotin-labeled tau (4.35 µM) immediately after the addition of heparin (1 µM) or vehicle (0 h), and again after 6 h. Values represent the change in ThS fluorescence (excitation 450 nm, emission 510 nm) over the 6 h period

To examine the elimination profile of exogenous tau from the brain, we evaluated the timecourse by which biotin-labeled tau is eliminated from the brain following intracranial injection. The half-life of exogenous biotin-labeled tau residing in the brain was 41 min (Fig. 1A), while the half-life of 10 kDa LyD, which does not readily cross the BBB, was nearly 3-times greater (114 min) (Fig. 1A). Next, the influence of r-mTBI on the elimination of exogenous tau species from the brain was evaluated after the intracranial injection of biotin-labeled tau (2 h). A two-way ANOVA evaluating the effect of r-mTBI and the tau species on tau residence time demonstrated a statistically significant interaction effect [*F*_(3,42)_ = 2.85, *p* < 0.05]. A significant main effect was observed with respect to injury [*F*_(1,42)_ = 12.23, *p* < 0.01] and the tau species [*F*_(3,42)_ = 7.18, *p* < 0.001]. Post hoc Bonferroni tests with multiple comparisons between r-mTBI and r-sham revealed a significant increase (*p* < 0.001) in monomeric tau residence in r-mTBI (2.62 ± 1.37) compared to r-sham (0.95 ± 0.59) (Fig. 1B). While the aggregate enriched species showed a twofold elevation in biotin-labeled tau residence time in the brain post-injury (1.25 ± 0.72) compared to r-sham (0.54 ± 0.20), this value did not reach statistical significance (*p* = 0.20) (Fig. 1B). Furthermore, additional post hoc Bonferroni tests with multiple comparisons evaluated each of the biotin-labeled tau species under r-mTBI conditions. There was no significant difference between the amount of exogenous monomeric and aggregated enriched species residing in the brain in the r-sham mice. In the r-mTBI mice, there was a significant (*p* < 0.001) decrease in the residence of seed competent tau (0.66 + 0.35) compared to the amount of exogenous monomeric tau (2.62 ± 1.37) (Fig. 1B). Similarly, a significant decrease (*p* < 0.01) in the residence of aggregate enriched tau (1.25 ± 0.72), relative to exogenous monomeric tau (2.62 ± 1.37) was observed (Fig. 1B). Of note, no statistically significant difference in the amount of dextran residing in the brain was observed between the r-mTBI and r-sham groups for any of the biotin-labeled tau species, indicating the effects of r-mTBI on tau elimination from the brain were not due to alterations in BBB integrity (data not shown). To characterize tau conformation status, MC1 immunoreactivity was elevated in seed competent and aggregate enriched biotin-labeled tau compared to monomeric biotin-labeled tau (Fig. 1C, D). The heparin-induced aggregated tau demonstrated substantial activity in the ThS assay, while biotin-labeled monomeric tau showed minimal activity, near the assay limit of detection (Fig. 1E).

### Tau uptake and caveolin-1 inhibition ex vivo

**Fig. 2:**
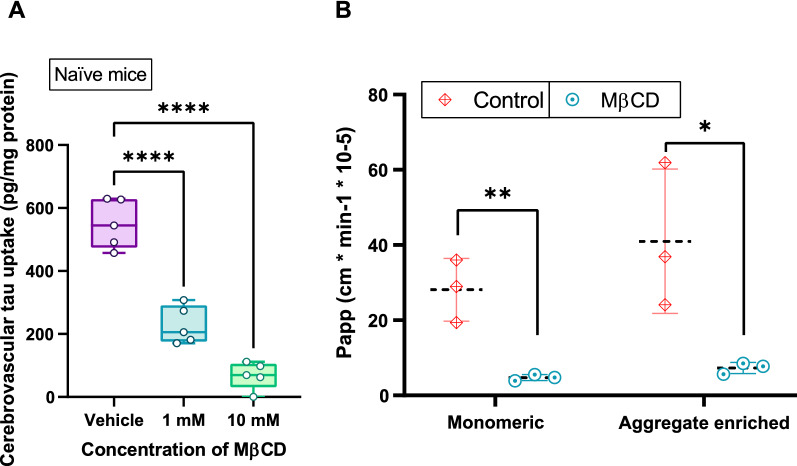
Effect of MβCD on tau transcytosis across an in vitro model of the BBB. **A** Freshly isolated cerebrovessels from naïve wild-type mice (9 months old) were pretreated with various concentrations of the caveolin inhibitor MβCD (0, 1 mM, 10 mM) for 30 min at 37 °C, before being exposed to recombinant human tau (5 ng/ml) for 1 h at 37 °C. Lysates were analyzed for tau content using an ELISA and normalized total protein using the BCA assay. Values represent mean ± SD (n = 5) and are expressed as pg of tau per mg of total protein. ****P < 0.0001 compared to vehicle as determined by one-way ANOVA and Bonferroni post-hoc test. **B** MβCD (10 mM) was exposed to the basolateral compartment of the in vitro BBB model for 30 min at 37 °C. Following the pretreatment with MβCD, biotin-labeled monomeric or aggregate enriched biotin-labeled tau was added alongside the known paracellular marker 10 kDa LyD to the basolateral compartment of the in vitro BBB model. Samples were collected from the apical compartment at 0, 30, and 60 min to determine the permeability of biotin-labeled tau and LyD across the BBB model. Values represent mean ± SD (n = 3) and are expressed as the apparent permeability coefficient (Papp). *P < 0.05, **P < 0.01 compared to each respective tau species with vehicle as determined by unpaired *t*-Test

Our previous work showed reduced cerebrovascular tau uptake coinciding with diminished caveolin-1 expression following r-mTBI (12 months post-injury) [45]. To further investigate the interaction between cerebrovascular caveolin-1 and extracellular tau, freshly isolated cerebrovessels from naïve mice were treated with methyl-β-cyclodextrin, a known modulator of caveolin-1 [28, 49], prior to exposure to unlabeled monomeric tau. A one-way ANOVA revealed a statistically significant treatment effect [*F*_(2,12)_ = 78.21, *p* < 0.0001, n = 5] on cerebrovascular tau uptake (Fig. 2A). Post hoc Bonferroni tests with multiple comparisons revealed a significant decrease (*p* < 0.0001) in tau uptake for both 1 mM MβCD (227.8 ± 59.83) and 10 mM MβCD (68.31 ± 43.23) when compared to untreated cerebrovascular tau uptake (549.8 ± 78.05).

### Tau transcytosis across an in vitro BBB model

To more specifically investigate the mechanism by which tau is eliminated from the brain, we examined tau transit across an in vitro model of the BBB. In comparing the basolateral-to-apical BBB transit of each biotin-labeled tau species, there were no differences in the apparent permeability of each biotin-labeled tau species across the BBB model under control conditions. Pre-treatment with MβCD resulted in a significant decrease (unpaired *t*-Test, *t*(4) = 4.830, *p* < 0.01, n = 3) in the BBB transcytosis of monomeric tau (4.75 ± 0.81) compared to untreated monomeric tau transcytosis (28.10 ± 8.34) (Fig. 2B). Similarly, pre-treatment with MβCD resulted in a significant decrease (unpaired *t*-Test, *t*(4) = 3.028, *p* < 0.05, n = 3) in aggregate enriched tau transcytosis (7.30 ± 1.48) compared to untreated aggregate enriched tau transcytosis (40.97 ± 19.20) (Fig. 2B). Notably, the individual biotin-labeled tau species did not appear to impact BBB integrity as dextran permeability across the BBB model was not different between the biotin-labeled tau species. The percentage of dextran crossing the BBB model over the 60 min period was 0.90 ± 0.09% for monomeric tau and 0.88 ± 0.1% for aggregate enriched tau. Likewise, treatment with MβCD had no effect on dextran BBB permeability compared to control conditions. The percentage of dextran crossing the BBB model over the 60 min period was 0.89 ± 0.04% for control and 0.84 ± 0.03% for MβCD.

### Cerebrovascular expression of caveolin-1 and Mfsd2a in r-mTBI animals

**Fig. 3:**
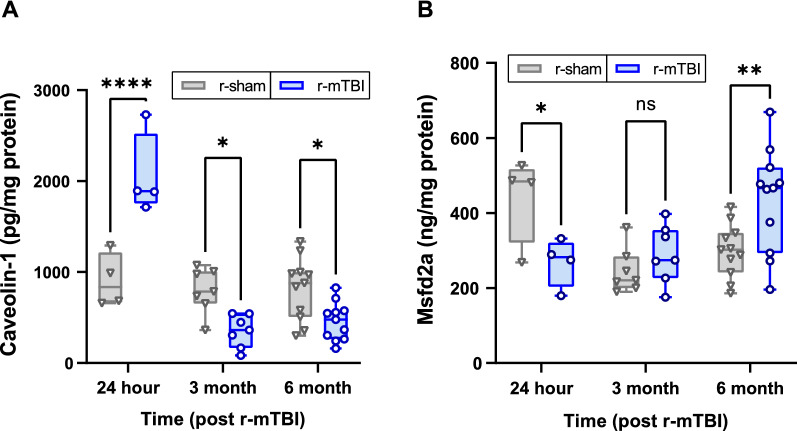
Effect of r-mTBI on caveolin-1 and Mfsd2a levels in freshly isolated cerebrovessels from r-mTBI hTau mice (24 h, 3 months, 6 months post-last injury). Lysates were analyzed for **A** caveolin-1 and **B** Mfsd2a by ELISA and normalized to total protein using the BCA assay. Values represent mean ± SD (24 h n = 4, 3 month n = 7, 6 month n = 11) and are expressed as pg of caveolin-1 or ng Mfsd2a per mg total protein. *P < 0.05, **P < 0.01, ****P < 0.0001 compared to each respective r-sham as determined by two-way ANOVA and Bonferroni’s multiple comparisons test

As Mfsd2a has been shown to regulate caveolin-1 expression, Mfsd2a and caveolin-1 levels were examined in isolated cerebrovessels from r-sham and r-mTBI animals. A two-way ANOVA was conducted that examined the effects of r-mTBI and time on cerebrovascular caveolin-1 expression which revealed a statistically significant interaction [*F*_(2,38)_ = 24.37, *p* < 0.0001] on caveolin-1 expression. A significant main effect was observed with respect to time [*F*_(2,38)_ = 30.00, *p* < 0.001], but there was no significant main effect with respect to injury [*F*_(1,38)_ = 1.496, *p* = 0.229]. Post hoc Bonferroni tests with multiple comparisons between r-sham and r-mTBI were conducted to evaluate whether r-mTBI could influence the expression of cerebrovascular caveolin-1 compared to r-sham animals at each time point. This revealed a significant increase (*p* < 0.001) in cerebrovascular caveolin-1 in r-mTBI animals (2055 ± 457.7) compared to r-sham animals (905.7 ± 298.3) at 24 h post injury (Fig. 3A). Alternatively, at 3 months post-injury, r-mTBI cerebrovessels (350.7 ± 178.3) showed a significant (*p* < 0.05) reduction in caveolin-1 (twofold) relative to r-sham animals (800.0 ± 248.0), and a similar significant (*p* < 0.05) reduction was seen at 6 months in r-mTBI cerebrovessels (457.6 ± 209.2) compared to r-sham (816.7 ± 340.7) (Fig. 3A). With respect to Mfsd2a, a two-way ANOVA evaluating the influence of r-mTBI and time post injury on Mfsd2a expression revealed a significant interaction [*F*_(2,38)_ = 7.482, *p* < 0.01] and a significant main effect with respect to time [*F*_(2,38)_ = 4.718, *p* < 0.05], but no significant main effect with respect to injury [*F*_(1,38)_ = 0.001, *p* = 0.92]. Post hoc Bonferroni tests with multiple comparisons between r-mTBI and sham revealed that at 24 h post injury, there was a significant decrease (*p* < 0.05) in cerebrovascular Mfsd2a in r-mTBI animals (268.9 ± 64.2) compared to sham animals (440.7 + 116.7) (Fig. 3B). At 3 months post injury, there was no significant difference (*p* = 0.39) between r-mTBI (291.0 ± 77.0) and r-sham (245.6 ± 59.9). However, at 6 months post injury there was a significant increase (*p* < 0.01) in cerebrovascular Mfsd2a in r-mTBI animals (434.9 ± 138.8) compared to r-sham (299.0 ± 70.9).

### Ang-1/Ang-2 secretion from r-mTBI cerebrovessels ex vivo

**Fig. 4:**
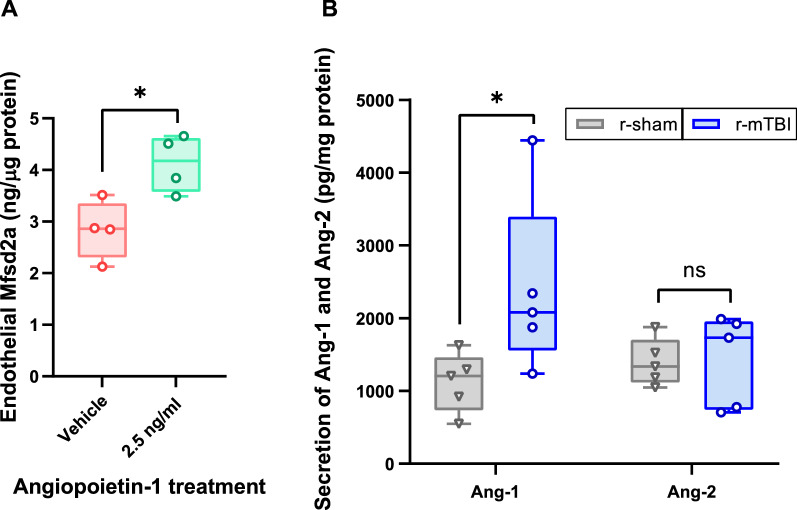
Influence of r-mTBI on cerebrovascular angiopoietin secretion. **A** Mfsd2a expression in HBMEC following treatment with Ang-1 (2.5 ng/ml) for 24 h at 37 °C. Ang-1 expression was quantified by ELISA and normalized to total protein using the BCA assay. Values represent mean ± SD (n = 4) and are expressed as ng Mfsd2a per μg of total protein. *P < 0.05 compared to vehicle as determined by unpaired *t*-Test. **B** Secretion of Ang-1 and Ang-2 from fresh cerebrovessels isolated from r-sham and r-mTBI hTau mice. Following 72 h of incubation at 37 °C, the cerebrovascular extracellular media was probed for Ang-1 and Ang-2 using an ELISA and normalized to total protein using the BCA assay. Values represent mean ± SD (n = 5) and are expressed as pg of Ang-1 or Ang-2 per mg total protein. *P < 0.05 compared to each respective r-sham as determined by a Mann–Whitney U test

Prior reporting has indicated the growth factor Ang-1, which is primarily secreted by vascular mural cells, was elevated in the acute stages after TBI [22] and associated with diminished caveolin-1 expression [41]. However, the influence of Ang-1 on Mfsd2a and caveolin-1 expression in the chronic stages post-injury has yet to be investigated. Administration of Ang-1 to HBMECs in vitro over 24 h lead to a significant increase in levels of Mfsd2a (4.12 ± 0.55) compared to untreated cells (2.84 ± 0.57) (unpaired *t*-Test, *t*(6) = 3.247, *p* < 0.05, n = 4) (Fig. 4A). At 6 months post injury, a Mann–Whitney test indicated that secreted Ang-1 levels from isolated r-mTBI cerebrovessels (*Mdn* = 2080) were significantly higher than r-sham (*Mdn* = 1206) over 72 h (*U* = 2, *p* < 0.05, n = 5) (Fig. 4B), while there was no significant difference in Ang-2 secretion between r-mTBI (*Mdn* = 1337) and r-sham (*Mdn* = 1731) (Fig. 4B) (*U* = 11, *p* = 0.841, n = 5).

## Discussion

As pathological tau propagation has been observed in the chronic stages of TBI and other neurodegenerative diseases, we explored potential mechanisms responsible for the elimination of extracellular tau from the brain and the influence of head trauma on these processes. Our recent work demonstrated an interaction between extracellular tau and brain vascular mural cells (pericytes and smooth muscle cells) and showed a progressive decrease in cerebrovascular tau uptake up to 12 months post-injury in our mouse r-mTBI model [45]. The reduced cerebrovascular tau uptake following r-mTBI coincided with a significant decrease in caveolin-1 levels in r-mTBI cerebrovessels compared to r-sham animals at 12 months post-injury [45]. The present studies continued this line of investigation to determine the mechanisms driving cerebrovascular tau elimination and the potential impact of head trauma on these processes.

The clearance of extracellular tau from the ISF has not been extensively characterized, though it appears that extracellular tau can readily enter the plasma as increases in ISF tau due to neuronal injury are reflected in the plasma shortly after injury [61]. After injection into the ISF, tau relocates the perivascular space within and around arteriole walls within minutes, and though it is not cleared as efficiently as Aβ, recent evidence suggests they share common routes of cerebrovascular elimination [43]. It was determined that the half-life of tau injected into the cisterna magma was less than 2 h and the exogenous tau was detectable in the plasma within minutes of the injection [61]. In line with these studies, our work found the half-life of exogenous tau was approximately 41 min in the brain following intracranial injection. For context, we also injected a 10 kDa dextran marker (LyD), that is not readily eliminated from the brain and does not cross the BBB [42], and found the half-life of LyD in the brain was 4-times the value we observed for exogenous tau (164 min vs. 41 min). Prior reporting has indicated a significantly longer half-life for tau in the brain, 11 days in mice [60] and 20 days in humans [52]. However, it is important to note these values encompassed the entire life cycle of tau, from neuronal synthesis and cellular secretion to elimination, whereas the half-life value for tau in the present study only reflects elimination from the brain. Thus, based on the elimination profile of tau from the brain in our studies, we used a 2-h post-injection time-point to examine the influence of head trauma on tau residence in the brain.

The amount of exogenous monomeric tau residing in the brain was greater in the r-mTBI animals compared to r-sham, indicating tau elimination from the brain was reduced following head trauma. Interestingly, the monomeric tau residing in the r-mTBI brain was greater than that observed for the seed competent and aggregate enriched tau species. While studies evaluating the interactions of various tau species with brain endothelia are lacking, earlier work suggested tau aggregates, but not monomeric tau, efficiently bind to neurons and are internalized using bulk endocytosis [58]. A more recent report found that both monomeric and aggregated tau can be internalized with similar efficiency in neurons, but may do so through distinct pathways [18]. Solute internalization and trafficking at the BBB are both regulated by ligand binding avidity and particle size [56]. which differ between tau aggregates and monomers, and may describe any differences in endothelial internalizing and trafficking amongst various tau species. Clearly, further work is necessary to understand the regulatory mechanisms governing cerebrovascular tau elimination and the potential influence of head trauma on these processes.

There are several pathways through which tau can be eliminated from the brain including, but not limited to, (1) degradation, (2) perivascular drainage, and (3) BBB transcytosis. While tau has been shown to be degraded in the brain, these processes generally occur over a longer period of time (> 12 h) [12, 14] than the 2–3 h elimination time-frame in our experimental paradigm, so degradation does not appear to be a primary driver of extracellular tau elimination from the brain in our particular studies. Previous work found that paravascular tau clearance was reduced by approximately 60% following TBI and was associated with phospho-tau pathology and neurodegeneration [26]. The authors noted that if there were a tau efflux mechanism, at the BBB for example, their theory of convective bulk flow may contribute to tau ISF clearance by effectively distributing tau along the vascular bed for more efficient transcytosis [26]. That said, there has been little investigation into the transit of tau across the BBB. Banks and colleagues found that full-length tau and various truncated tau proteins readily crossed the BBB bidirectionally and entered the blood following injection into the brain [8], consistent with our intracranial tau injection studies. In line with these BBB findings, our studies employed a recently described indirect co-culture BBB model [30] consisting of brain endothelia and pericytes, and found the BBB transit of each tau species was greater than that observed for LyD (basolateral-to-apical). In comparing the tau species, the aggregate enriched tau showed increased BBB transcytosis compared to monomeric tau, potentially explaining the enhanced elimination of these species in the tau residence studies above.

As indicated above, our prior work showed a correlation between tau uptake and caveolin-1 expression in isolated cerebrovessels, suggesting tau endocytosis in these cells may be mediated by caveolin-1. In the present studies, modulating caveolae with MβCD resulted in a significant decrease in cerebrovascular tau uptake and dramatically reduced the BBB transit of both tau monomers and aggregate enriched species of tau. Of note, while several studies have demonstrated MβCD to be an effective disruptor of caveolae and caveolin-1 [28, 49] we cannot rule out any nonspecific effects of MβCD (i.e., other than caveolin-1) on cerebrovascular tau transit. That said, it was previously reported that mice with reduced caveolin-1 brain expression showed elevated levels of total and phosphorylated tau in the hippocampus compared to wild-type animals [10, 25]. Collectively these findings indicate tau proteins can be eliminated across the BBB, which may be mediated through caveolin-1.

There is a dearth of research into the long-term effects of TBI on caveolin-1, particularly in the context of repetitive injury, but our previous studies found a significant downregulation in cerebrovascular caveolin-1 levels at 12 months post-injury in a mouse r-mTBI model [45]. Looking more acutely, our current results demonstrate an upregulation of caveolin-1 expression at 24 h post-injury, consistent with prior reporting of increased caveolin-1 expression within the neurovascular unit during the same 24-h period following TBI in juvenile rats [6]. However, in this rat TBI model, the increased expression appeared to be transient, as caveolin-1 levels returned to baseline 3 days after the brain injury [6]. The acute upregulation of caveolin-1 post-injury could explain the transient increase in plasma tau levels observed immediately following sports-related concussive injury [21]. In the more chronic phase post-injury, we observed decreased expression of caveolin-1 following r-mTBI at 3 and 6 months post-injury compared to r-sham, consistent with what was observed at 12 months post-injury [45]. It has been proposed that the upregulation of caveolin-1 acutely following cerebral insult is meant to facilitate vascular repair by promoting angiogenesis and stabilizing tight junction and efflux proteins (e.g., claudin-5 and P-glycoprotein) [6]. However, as observed in the present studies, the chronic downregulation of caveolin-1 after r-mTBI could hamper waste elimination from the brain, leading to the accumulation of extracellular solutes such as tau, as demonstrated in other animal models where caveolin-1 is chronically diminished in the brain [10, 25].

A primary regulator of caveolae-mediated transcytosis in brain endothelia is the lipid transporter Mfsd2a, which inhibits caveolae vesicle formation by modulating the lipid composition of brain endothelial membranes to suppress transcytosis [2] and regulate BBB integrity [62]. As a result, transcytosis via caveolin-1 is inversely related to Mfsd2a expression in the BBB. Correspondingly, we found cerebrovascular Mfsd2a levels were decreased at 24 h following r-mTBI, while caveolin-1 levels were significantly increased compared to r-sham animals during this time frame. Along these lines, prior reporting showed Mfsd2a levels were significantly decreased in the acute stage after surgical brain injury [16, 17] and, interestingly, Mfsd2a upregulation was found to reverse BBB disruption by altering caveolae-based transport, providing neuroprotection following injury-induced sub-arachnoid hemorrhage [63]. In terms of the chronic phase post-injury, there is a lack of evidence regarding the response of cerebrovascular Mfsd2a to head trauma. That said, while Mfsd2a levels are generally decreased in acute conditions such as intracerebral hemorrhage, Mfsd2a has been shown to be largely upregulated in more chronic disorders such as chronic liver injury and inflammatory bowel disease [16]. In line with these studies, we observed a progressive increase in cerebrovascular Mfsd2a levels in the chronic phase post-injury (6 months following r-mTBI), though more work is necessary to understand the consequence of altered Mfsd2a expression in the brain following head trauma, especially in the more chronic stages post-injury.

Mfsd2a expression in brain endothelia has been shown to be directly regulated by cerebrovascular mural cells [9]. Dysfunctional endothelial transcytosis after injury appears to be primarily driven by altered pericyte crosstalk with endothelial cells [54]. To this point, the mechanism by which mural cells regulate endothelial expression of Mfsd2a and caveolin-1 is unclear, though evidence suggests the signaling factors angiopoietin-1 (Ang-1) and angiopoietin-2 (Ang-2) may play a role. Ang-1 is predominantly expressed and constitutively released by vascular mural cells [20, 55]. to facilitate vessel assembly and stability and is a critical survival factor in preventing endothelial cell death [29]. In addition, Ang-1 is known to activate the signaling pathway that downregulates plasmalemma vesicle-associated protein expression, which is also responsible for caveolin formation [31]. Alternatively, Ang-2 is almost exclusively secreted by endothelial cells and functions as a negative regulator of the Ang-1 pathway in order to modulate vessel maturation [19, 24]. The impact of Ang-1 on caveolin-1 expression was demonstrated recently in rats, where increased caveolin-1 levels following acute brain trauma were reversed with Ang-1 treatment [41]. In a similar manner, our studies found that treatment with Ang-1 significantly increased Mfsd2a expression in human brain endothelial cells. Altogether, alterations in the secretion of Ang-1 (from brain vascular mural cells) or Ang-2 (from brain endothelia) following brain injury could influence endothelial transcytosis by modulating the Mfsd2a/caveolin-1 pathway.

Previous reports have shown vascular mural cells upregulate Ang-1 in response to insults such as hypoxic conditions, while Ang-2 levels were largely decreased or unchanged [15, 48]. With respect to TBI, prior studies have reported a progressive decrease in Ang-1 levels in the brain [50] and capillaries [15] over the first 48 h following brain injury. At later stages of brain injury, pericytes become reactive and secrete angiogenic growth factors including Ang-1 to mediate endothelial cell activity and vascular integrity [51]. There has been a lack of work examining Ang-2 levels following TBI, however Ang-2 levels were found to be increased acutely in the brain following subarachnoid hemorrhage [23] and cold-injury [44], but the chronic status of Ang-2 in the brain following cerebral insult is currently unknown. That said, as the time increases post-injury (days and weeks following head trauma), several studies have reported an increase in Ang-1 levels in the brain [11, 50] and serum [22]. Our studies coincide with these findings as freshly isolated cerebrovessels from r-mTBI animals (6 months post-injury) secreted significantly higher levels of Ang-1 compared to r-sham animals, while cerebrovascular Ang-2 secretion was unchanged between the r-mTBI and r-sham animals.

## Conclusions

These studies indicate tau elimination from the brain is diminished following r-mTBI, which may be the result of decreased caveolin-1-mediated tau transit across the BBB. Our prior work showed mural cell dysfunction and decreased cerebrovascular tau uptake coinciding with reduced caveolin-1 expression in the chronic phase following r-mTBI. The current studies suggest the changes in caveolin-1 post-injury may be due to alterations in Mfsd2a expression in the BBB in response to increased Ang-1 secretion from dysfunctional mural cells in the aftermath of brain injury. Taken together, aberrant mural cell function following r-mTBI diminishes caveolae-mediated tau elimination across the BBB, which may describe the chronic accumulation of tau deposits typically observed in the brain following head trauma.

## Data Availability

The datasets from the current study can be made available upon reasonable request.

## References

[1] Andorfer C (2003). Hyperphosphorylation and aggregation of tau in mice expressing normal human tau isoforms. J Neurochem.

[2] Andreone BJ (2017). Blood-brain barrier permeability is regulated by lipid transport-dependent suppression of caveolae-mediated transcytosis. Neuron.

[3] Artursson P (1990). Epithelial transport of drugs in cell culture. I: a model for studying the passive diffusion of drugs over intestinal absorbtive (Caco-2) cells. J Pharm Sci.

[4] Bachmeier C (2014). Apolipoprotein E isoform-specific effects on lipoprotein receptor processing. NeuroMol Med.

[5] Bachmeier C, Mullan M, Paris D (2010). Characterization and use of human brain microvascular endothelial cells to examine β-amyloid exchange in the blood-brain barrier. Cytotechnology.

[6] Badaut J (2015). Caveolin expression changes in the neurovascular unit after juvenile traumatic brain injury: signs of blood-brain barrier healing?. Neuroscience.

[7] Banks WA (2015). The blood-brain barrier in neuroimmunology: tales of separation and assimilation. Brain Behav Immun.

[8] Banks WA (2016). Tau proteins cross the blood-brain barrier. J Alzheimer’s Dis.

[9] Ben-Zvi A (2014). Mfsd2a is critical for the formation and function of the blood-brain barrier. Nature.

[10] Bonds JA (2019). Depletion of caveolin-1 in Type 2 diabetes model induces Alzheimer’s disease pathology precursors. J Neurosci.

[11] Brickler TR (2018). Angiopoietin/tie2 axis regulates the age-at-injury cerebrovascular response to traumatic brain injury. J Neurosci.

[12] David DC (2002). Proteasomal degradation of tau protein. J Neurochem.

[13] Deane R (2009). Endothelial protein C receptor-assisted transport of activated protein C across the mouse blood-brain barrier. J Cereb Blood Flow Metab.

[14] Dolan PJ, Johnson GVW (2010). A caspase cleaved form of tau is preferentially degraded through the autophagy pathway. J Biol Chem.

[15] Dore-Duffy P (2007). Differential expression of capillary VEGF isoforms following traumatic brain injury. Neurol Res.

[16] Eser Ocak P, Ocak U, Sherchan P, Zhang JH (2020). Insights into major facilitator superfamily domain-containing protein-2a (Mfsd2a) in physiology and pathophysiology. What do we know so far?. J Neurosci Res.

[17] Eser Ocak P, Ocak U, Sherchan P, Gamdzyk M (2020). Overexpression of Mfsd2a attenuates blood brain barrier dysfunction via Cav-1/Keap-1/Nrf-2/HO-1 pathway in a rat model of surgical brain injury. Exp Neurol.

[18] Evans LD (2018). Extracellular monomeric and aggregated tau efficiently enter human neurons through overlapping but distinct pathways. Cell Rep.

[19] Felcht M (2012). Angiopoietin-2 differentially regulates angiogenesis through TIE2 and integrin signaling. J Clin Investig.

[20] Gaengel K (2009). Endothelial-mural cell signaling in vascular development and angiogenesis. Arterioscler Thromb Vasc Biol.

[21] Gill J (2017). Acute plasma tau relates to prolonged return to play after concussion. Neurology.

[22] Gong D (2011). Dynamic changes of vascular endothelial growth factor and angiopoietin-1 in association with circulating endothelial progenitor cells after severe traumatic brain injury. J Trauma Inj Infect Crit Care.

[23] Gu H (2016). Angiopoietin-1 and angiopoietin-2 expression imbalance influence in early period after subarachnoid hemorrhage. Int Neurourol J.

[24] Hansen TM (2010). Effects of angiopoietins-1 and -2 on the receptor tyrosine kinase Tie2 are differentially regulated at the endothelial cell surface. Cell Signal.

[25] Head BP (2010). Loss of caveolin-1 accelerates neurodegeneration and aging. PLoS ONE.

[26] Iliff JJ (2014). Impairment of glymphatic pathway function promotes tau pathology after traumatic brain injury. J Neurosci.

[27] Jicha GA (1997). Alz-50 and MC-1, a new monoclonal antibody raised to paired helical filaments, recognize conformational epitopes on recombinant tau. J Neurosci Res.

[28] Jozic I (2019). Pharmacological and genetic inhibition of caveolin-1 promotes epithelialization and wound closure. Mol Ther.

[29] Kim I (2000). Angiopoietin-1 regulates endothelial cell survival through the phosphatidylinositol 3’-kinase/Akt signal transduction pathway. Circ Res.

[30] Kurmann L, Okoniewski M, Dubey RK (2021). Transcryptomic analysis of human brain -microvascular endothelial cell driven changes in -vascular pericytes. Cells.

[31] Laksitorini MD (2019). Modulation of Wnt/β-catenin signaling promotes blood-brain barrier phenotype in cultured brain endothelial cells. Sci Rep.

[32] Magnoni S (2012). Tau elevations in the brain extracellular space correlate with reduced amyloid-β levels and predict adverse clinical outcomes after severe traumatic brain injury. Brain.

[33] Marklund N (2009). Monitoring of brain interstitial total tau and beta amyloid proteins by microdialysis in patients with traumatic brain injury: clinical article. J Neurosurg.

[34] McKee AC (2009). Chronic traumatic encephalopathy in athletes: progressive tauopathy after repetitive head injury. J Neuropathol Exp Neurol.

[35] McKee AC (2016). The first NINDS/NIBIB consensus meeting to define neuropathological criteria for the diagnosis of chronic traumatic encephalopathy. Acta Neuropathol.

[36] Medina M, Avila J (2014). The role of extracellular Tau in the spreading of neurofibrillary pathology. Front Cell Neurosci.

[37] Mirbaha H (2018). Inert and seed-competent tau monomers suggest structural origins of aggregation. Elife.

[38] Motulsky HJ.“Confidence intervals of parameters,” GraphPad curve fitting guide. 2016. http://www.graphpad.com/guides/prism/7/curve-fitting/index.htm?reg_standard_errors_and_confidence.htm. Accessed 1 May 2021.

[39] Mouzon B (2012). Repetitive mild traumatic brain injury in a mouse model produces learning and memory deficits accompanied by histological changes. J Neurotrauma.

[40] Mouzon BC (2014). Chronic neuropathological and neurobehavioral changes in a repetitive mild traumatic brain injury model. Ann Neurol.

[41] Nag S (2017). Molecular changes associated with the protective effects of angiopoietin-1 during blood-brain barrier breakdown post-injury. Mol Neurobiol.

[42] Natarajan R, Northrop N, Yamamoto B (2017). Fluorescein isothiocyanate (FITC)-dextran extravasation as a measure of blood-brain barrier permeability. Curr Protoc Neurosci.

[43] Nimmo J (2020). Peri-arterial pathways for clearance of α-Synuclein and tau from the brain: Implications for the pathogenesis of dementias and for immunotherapy. Alzheimer’s Dement Diagn Assess Dis Monit.

[44] Nourhaghighi N (2003). Altered expression of angiopoietins during blood-brain barrier breakdown and angiogenesis. Lab Invest.

[45] Ojo J (2021). Mural cell dysfunction leads to altered cerebrovascular tau uptake following repetitive head trauma. Neurobiol Dis.

[46] Öst M (2006). Initial CSF total tau correlates with 1-year outcome in patients with traumatic brain injury. Neurology.

[47] Paris D (2011). Selective antihypertensive dihydropyridines lower Aβ accumulation by targeting both the production and the clearance of Aβ across the blood-brain barrier. Mol Med.

[48] Park YS (2016). Expression of angiopoietin-1 in hypoxic pericytes: regulation by hypoxia-inducible factor-2α and participation in endothelial cell migration and tube formation. Biochem Biophys Res Commun.

[49] Potje SR (2019). Reduced caveolae density in arteries of SHR contributes to endothelial dysfunction and ROS production. Sci Rep.

[50] Sabirzhanov B (2018). MicroRNA-711-Induced downregulation of angiopoietin-1 mediates neuronal cell death. J Neurotrauma.

[51] Salehi A, Zhang JH, Obenaus A (2017). Response of the cerebral vasculature following traumatic brain injury. J Cereb Blood Flow Metab.

[52] Sato C (2018). Tau kinetics in neurons and the human central nervous system. Neuron.

[53] Sui YT (2014). Alpha synuclein is transported into and out of the brain by the blood-brain barrier. Peptides.

[54] Sun Z (2021). Reduction in pericyte coverage leads to blood–brain barrier dysfunction via endothelial transcytosis following chronic cerebral hypoperfusion. Fluids Barriers CNS.

[55] Sundberg C (2002). Stable expression of angiopoietin-1 and other markers by cultured pericytes: phenotypic similarities to a subpopulation of cells in maturing vessels during later stages of angiogenesis in vivo. Lab Invest.

[56] Tian X (2020). On the shuttling across the blood-brain barrier via tubule formation: Mechanism and cargo avidity bias. Sci Adv.

[57] Wang J (2018). Physiological clearance of tau in the periphery and its therapeutic potential for tauopathies. Acta Neuropathol.

[58] Wu JW (2013). Small misfolded tau species are internalized via bulk endocytosis and anterogradely and retrogradely transported in neurons. J Biol Chem.

[59] Yamada K (2011). In vivo microdialysis reveals age-dependent decrease of brain interstitial fluid tau levels in P301S human tau transgenic mice. J Neurosci.

[60] Yamada K (2015). Analysis of in vivo turnover of tau in a mouse model of tauopathy. Mol Neurodegener.

[61] Yanamandra K (2017). Anti-tau antibody administration increases plasma tau in transgenic mice and patients with tauopathy. Sci Transl Med.

[62] Yang YR (2017). Mfsd2a (major facilitator superfamily domain containing 2a) attenuates intracerebral hemorrhage-induced blood-brain barrier disruption by inhibiting vesicular transcytosis. J Am Heart Assoc.

[63] Zhao C (2020). Mfsd2a attenuates blood-brain barrier disruption after sub-arachnoid hemorrhage by inhibiting caveolae-mediated transcellular transport in rats. Transl Stroke Res.

